# Follow-Up of Peripheral IL-1β and IL-6 and Relation with Apoptotic Death in Drug-Resistant Temporal Lobe Epilepsy Patients Submitted to Surgery

**DOI:** 10.3390/bs8020021

**Published:** 2018-02-05

**Authors:** Lourdes Lorigados Pedre, Lilia M. Morales Chacón, Nancy Pavón Fuentes, María de los A. Robinson Agramonte, Teresa Serrano Sánchez, Rachel M. Cruz-Xenes, Mei-Li Díaz Hung, Bárbara Estupiñán Díaz, Margarita M. Báez Martín, Sandra Orozco-Suárez

**Affiliations:** 1 Immunochemical Department, International Center for Neurological Restoration, 25th Ave, Playa, 15805, PC 11300 Havana, Cuba; nancy@neuro.ciren.cu (N.P.F.); robin@neuro.ciren.cu (M.d.l.A.R.A.); teresa@neuro.ciren.cu (T.S.S.); mdiazhung@gmail.com (M.L.D.H); 2 Clinical Neurophysiology Lab., International Center for Neurological Restoration, PC 11300 Havana, Cuba; lily@neuro.ciren.cu (L.M.M.C.); minou@neuro.ciren.cu (M.M.B.M.); 3 Biology Faculty, University of Havana, PC 10400 Havana, Cuba; rachel.cruz@fbio.uh.cu; 4 Morphological Laboratory, International Center for Neurological Restoration, PC 11300 Havana, Cuba; baby@neuro.ciren.cu; 5 Unit of Medical Research in Neurological Diseases, Specialty Hospital, National Medical Center, XXI Century IMSS, PC 06720 Mexico City, Mexico; sorozco5@hotmail.com

**Keywords:** drug-resistant temporal lobe epilepsy, inflammation, apoptosis, IL-1β, IL-6, NF-κB

## Abstract

Increasing amounts of evidence support the role of inflammation in epilepsy. This study was done to evaluate serum follow-up of IL-1β and IL-6 levels, as well as their concentration in the neocortex, and the relationship of central inflammation with NF-κB and annexin V in drug-resistant temporal lobe epileptic (DRTLE) patients submitted to surgical treatment. Peripheral and central levels of IL-1β and IL-6were measured by ELISA in 10 DRTLE patients. The sera from patients were taken before surgery, and 12 and 24 months after surgical treatment. The neocortical expression of NF-κB was evaluated by western blotting and annexin V co-localization with synaptophysin by immunohistochemistry. The neocortical tissues from five patients who died by non-neurological causes were used as control. Decreased serum levels of IL-1 and IL-6 were observed after surgery; at this time, 70% of patients were seizure-free. No values of IL-1 and IL-6 were detected in neocortical control tissue, whereas cytokine levels were evidenced in DRTLE. Increased NF-κB neocortex expression was found and the positive annexin V neurons were more obvious in the DRTLE tissue, correlating with IL-6 levels. The follow-up study confirmed that the inflammatory alterations disappeared one year after surgery, when the majority of patients were seizure-free, and the apoptotic death process correlated with inflammation.

## 1. Introduction

Epilepsy is a chronic brain disease that affects around 50 million people worldwide. In a high number of cases it is characterized by the presence of recurrent seizures, which are the result of an excessive electrical discharge of a neuronal group in a certain part of the brain [1]. The battery of treatments designed to counteract the clinical manifestations of this disease is diverse and ranges from a wide spectrum of antiepileptic drugs, specific diets, and sports-based therapies, to surgical techniques for resection of the epileptogenic focus [2]. Despite many efforts to find a successful pharmacological treatment for this condition, 30% of the population of patients with epilepsy cannot control the onset of seizures [3]. This clinical condition is known as drug-resistant epilepsy (DRE) or intractable epilepsy [4]. The literature has reported that temporal lobe epilepsy (TLE) shows one ofthe highest incidences of DRE [5]. Surgical treatment is currently an option for patients with drug-resistant temporal lobe epilepsy (DRTLE) and is the only treatment that clearly shows a positive action in decreasing the frequency of seizures and the progression of the disease in these patients [6].

Several authors suggest the contribution of immunological and inflammatory mechanisms in the pathogenesis of epilepsy based on the favorable effects of treatment with intravenous immunoglobulins, corticosteroids, and anti-inflammatories [7,8,9,10]. The immunological alterations described in epilepsy are associated, in most cases, with anti-epileptic drug treatment [11,12]; in others, they have not been related to pharmacotherapy [13,14]. Additionally, our group has described that immunological disorders in patients with epilepsy are associated with certain locations of the epileptogenic zone. Temporal lobe localization of the epileptogenic zone has been shown to be related to alterations in cellular immunity, and this dysfunction is not associated with pharmacological antiepileptic treatment [15,16]. However, currently, it has not been clarified whether inflammatory and immunological disorders are cause or consequence of the seizures in DRTLE.

Inflammation is considered an important factor in the pathophysiology of seizures. All the risk factors for epilepsy such as traumas, tumors, and infections are accompanied by different degrees of inflammation in the central nervous system, which is associated with the occurrence of seizures [17,18,19]. However, little is known about the contribution of peripheral inflammation on the modulation of these events as well as on the relationship of the neuronal loss described in epilepsy with inflammatory processes.

Proinflammatory cytokines are highly studied markers in both patients and experimental models of drug-resistant epilepsy. IL-1 and IL-6 are two of the most commonly approached proinflammatory cytokines in studies conducted to evaluate inflammation in drug-resistant epilepsy [20,21,22,23,24,25,26]. However, as far as we know, follow-up studies of the concentrations of inflammatory markers have not been described for a period of up to 2 years after removal of the epileptogenic focus by surgical techniques (standard lobectomies guided by electrocorticography).

The main objective of this work is to evaluate the proinflammatory proteins after the elimination of the epileptogenic focus and its relation with the neuronal loss described in patients with DRTLE.

## 2. Materials and Methods

### 2.1. Patient Information

Ten patients from the International Center for Neurological Restoration (CIREN) with DRTLE (6 female and 4 male, with mean age 33.1 ± 6.35 years) who underwent epilepsy surgery were included for the study of serum concentration of inflammatory cytokines (IL-1β and IL-6). The criteria to be considered drug-resistant were the following: present seizures for more than two years, two complex partial monthly seizures, use of two first-line anti-epileptic drugs, two cycles of monotherapy and at least one of polytherapy.

All patients were evaluated in the Video-EEG Telemetry Unit from CIREN, and they underwent a complete general and neurological physical examination and anatomical evaluation by Magnetic Resonance Imaging (1.5 T MAGNETOM SINPHONY) and SPECT.

Temporal localization presents unilateral rhythmic unloading with maximum amplitude in the zygomatic electrodes and in the anterior or middle temporal ones as the first electrographic change.

Antiepileptic treatment: The most commonly used drug was Carbamazepine (3–6 tablets daily) followed by Clobazan (3–4 tablets daily), Magnesium Valproate (1–6 tablets daily), and Lamotrigine (2–3 tablets daily). Up to two years after the surgery, all patients received the same drug treatment.

The neocortex adjacent to the hippocampus of the patients was resected at the time of surgery and the resection of the epileptic zone was performed by means of standard temporal lobectomies adjusted by electrocorticography.

The neocortical tissues from patients in this study were evaluated to determine the concentrations of IL-1β and IL-6, co-localization of annexinV and synaptophysine, and the expression of NF-κB. The neocortex control tissues were obtained from subjects who died due to non-neurological causes (Department of Pathological Anatomy from Clinical Hospital, Havana, Cuba). The control tissues were matched in age and gender with the group of DRTLE patients (average age: 35.2 years, 3 male and 2 female). They did not show macro- or microscopic pathological abnormalities, and the mean time of obtaining tissue was 3.2 h after the subject died.

### 2.2. Samples

The collected clinical parameters included demographic characteristics and clinical state showed in Table 1. The parameters were age; gender; personal pathological history; side of focus; disease duration; and the clinical state of patients, according to Engel’s scale, one and two years after surgery. Post-surgical seizure outcome assessment was based on the system proposed by Engel. (Engel class I, free of disabling seizures; class IA, seizure-free; class II, rare seizures (fewer than three seizures per year); class III, worthwhile improvement (reduction in seizures of 80% or more); class IV, no benefit [27].)

Blood: Venous Blood samples (5 mL) of the patients were taken by antecubital puncture, after asepsis of the region. The blood was centrifuged and the serum was kept at −20 °C until use. The samples of serum were obtained before and after surgical treatment (1 and 2 years of surgical evolution time).

Neocortical tissue: The neocortical tissue from patients with DRTLE was obtained during surgery. The tissue was washed with 0.9% saline solution at 4 °C, a fragment of approximately 0.5 cm^3^ was cut, placed in liquid nitrogen, and later transferred to a −80 °C freezer where it was preserved until use.

The neocortical control tissue was obtained from necropsy and was subsequently fractionated by areas. A fragment of approximately 0.5 cm^3^ was taken from the neocortex; this fragment was processed in a similar way to patient’s tissues.

Control samples were obtained from five subjects who died by vehicle accident (*n* = 4) and pulmonary thromboembolism (*n* = 1), and without history of neurological disease (Table 1). The neocortical tissue was dissected at the time of autopsy with a postmortem interval (PMI) from 2.5 to 4 h after death, and the samples were immediately stored at −70 °C. Previous works that used autopsy tissues with a PMI longer than the samples of the present work have shown the preservation of the protein and mRNA [28,29,30].

Homogenization of brain tissue: Tissue samples were homogenized in a glass–Teflon (Potter-Elvehjem), containing lysis buffer as described by the manufacturer (ENZO Life Sciences, Llorach, Germany)for the cytokine ELISA and 1mL of Trizol reagent (Invitrogen, Carlsbad, CA, USA) for the western blot. Protein was isolated following the manufacturer’s instructions.

### 2.3. Immunoenzymatic Assay for IL-1β and IL-6 in Serum and Brain Tissue

The concentrations of IL-1β and IL-6 (ENZO Life Sciences, Llorach, Germany) were measured by ELISA according to manufacturer’s instructions. The concentrations of IL-1β and IL-6 from the homogenate of the neocortical tissue of patients with DRTLE and control subjects was measured by the same methods. The lower limit of detection was 6pg/mL for IL-6 and 1 pg/mL for IL-β. Briefly, serum or homogenate from neocortical tissue was incubated in coated 96-well plates at room temperature for 2 h. The serum and tissue samples were applied in duplicate as well as the dilutions from the standard curves (IL-1β and IL-6). Plates were washed and then incubated with the detection antibody. After rewashing the plates, the conjugate was added for 30 min, followed by the substrate solution. The reaction was stopped with 1N H_2_SO_4_ and optical densities were measured at 450 nm using a microplate reader (ELx800 BioTek Instruments, Inc., Winooski, VT, USA).

### 2.4. Extraction of Neocortical Tissue Proteins and Western Blot to Evaluate NF-κB

The expression levels of NF-κB in the homogenates were analyzed by gel electrophoresis and western blot. The concentration of total proteins was determined by Lowry's method [31]. The absorbance values at 630 nm were obtained by spectrophotometry (BioTek Instruments, Winooski, VT, USA). Subsequently, the samples were separated by electrophoretic methods in SDS polyacrylamide gel. The proteins were transferred by electrophoresis to vinylidene membranes (Sigma-Aldrich, St. Louis, MO, USA). The membranes were blocked with 2% milk in phosphate buffer solution/Triton X-100 to avoid nonspecific binding. After blocking, the membranes were incubated individually with anti-NF-κB antibody and rabbit polyclonal anti-GAPDH (Glyceraldehyde-3-Phosphate Dehydrogenase, Santa Cruz Biotechnology, Santa Cruz, CA, USA) overnight with mild shaking at 4 °C (BrinkmannOrbMix 110, Brinkmann, Germany). The membranes were incubated for 2 h at room temperature in phosphate buffer containing goat anti-mouse IgG/rabbit conjugated with horseradish peroxidase diluted 1:1000 (Cell Signaling, Oregon City, OR, USA) for 1 h. The immunoreactive bands were detected by improved chemiluminescence. The densities of the protein signals in the films were quantified using ImageJ software.

To determine the integrity of the extracted proteins, 1D SDS-PAGE electrophoresis (12%) was used, followed by staining with Coomassie blue and western blot to identify the constitutive protein, indicating that there is no protein degradation.

### 2.5. Immunohistochemistry to Annexin V and Synaptophysin

The fragment of neocortical tissue obtained during surgery was washed with 0.9% saline at 4 °C and fixed in phosphate solution containing 4% paraformaldehyde and 0.2% glutaraldehyde. Subsequently, they were submerged in increasing solutions of sucrose (15, 20, 25, and 30%) and frozen at −70 °C. The neocortex tissue was cut into sections of 10 μm (Cryostat Leitz, 1720, Wetzlar, Germany). The pieces of tissue were blocked and incubated with the apoptotic marker annexin V and neuronal marker synaptophysin (Santa Cruz Biotechnology, Santa Cruz, CA, USA) according to the instructions of the manufacturer. An antibody conjugated with FITC (Zymed Laboratories Inc., San Francisco, CA, USA) was used for the immunodetection of antibodies against annexin V, while the immunodetection of synaptophysin was carried out by means of an antibody conjugated with Alexa Fluor 647 (Lab. Mol. Probes, Oregon City, OR, USA). The co-localization of the apoptotic and neuronal markers was evaluated in each patient’s tissue. The nuclei were visualized by counterstaining with propidium iodide. All sections were examined in a confocal microscope (Bio-Rad, Cambridge, UK) and each plate was examined by two specialists (SO and LL).

Quantitative Analysis: Only the sections that had consistent immunostaining were counted and 5 to 10 sections of each patient or control were quantified. The percentage of immunopositive cells to each marker (number of immunoreactive cells per mm^3^) was calculated in relation to the number of cells stained with propidium iodide per mm^3^, and the result was expressed as the percentage of cells positive for each marker. All sheets were examined by two specialists (SO and LL).

### 2.6. Ethical Considerations

All procedures followed the rules of the Declaration of Helsinki of 1975 for human research, and the study was approved by Record 03/2015, given by the Ethics Committee of the International Center for Neurological Restoration, named according to RESOLUTION No. 29-P/2011, on 24 October 2011. Each patient or family gave their informed consent.

### 2.7. Statistical Processing

Statistical analysis was carried out using the GraphPad Prism 5 software (GraphPad Software, Inc., La Jolla, CA, USA). The values are expressed as mean ± SEM. Normal distribution and homogeneity of variance of the data were tested by the Kolmogorov–Smirnov and Levene tests, respectively. The comparisons between two groups were made by means of the *t*-Student test while comparisons between more than two groups were made by one-way ANOVA, with post hoc Dunnet test. The Pearson correlation was used for the correlation study. In all cases, statistically significant differences were considered when *p* ≤ 0.05.

## 3. Results

### 3.1. Effect of Surgical Treatment in DRTLE Patients on the Peripheral (Serum) and Central (Neocortical Tissue) Concentrationsof IL-1βand IL-6.

**Figure 1:**
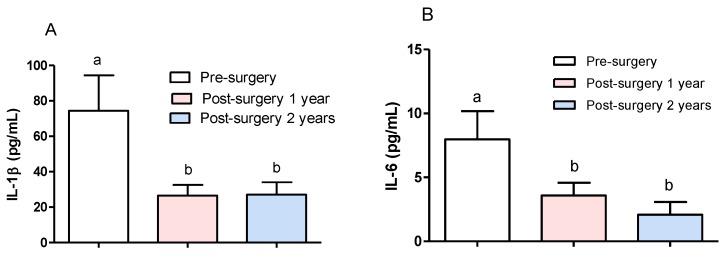
Comparison of IL-1β and IL-6 concentrations before and after surgical treatment. (**A**) IL-1β serum concentration. (**B**) IL-6 serum concentration. The bars represent the mean ± the standard error of the mean. One way ANOVA, post hoc Dunnet test, *p* ≤ 0.05.

**Figure 2:**
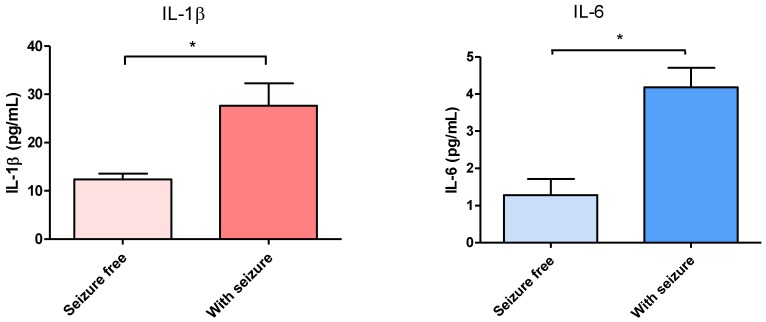
Concentration in serum of Interleukins according to the presence or absence of seizures one year after surgery. (**A**) IL-1β. (**B**) IL-6. The bars represent the mean ± the standard error of the mean. Mann Whitney test, * *p* ≤ 0.048.

In order to evaluate the effect on serum concentrations of IL-1β and IL-6 in DRTLE patients who received surgical treatment, we measured the concentrations of these cytokines before, and one and two years after the resection of the epileptogenic zone. In this study, there are no gender differences inboth levels of interleukins (IL-1 and IL-6), so the concentrations in male and female sexes were unified in one group. The results showed a statistically significant decrease of both (IL-1β and IL-6) cytokines (Figure 1A,B)one and two years after surgery evolution. In the first post-surgical evaluation, 70% of patients were in category IA according to the Engel scale (seizure-free), and those remaining had a significant decrease in seizure frequency (Table 2). One year after surgery treatment, lower values of IL-1β and IL-6 were observed in seizure-free patients, while patients who remained with seizures showed higher values of both cytokines (Figure 2A,B).

**Figure 3:**
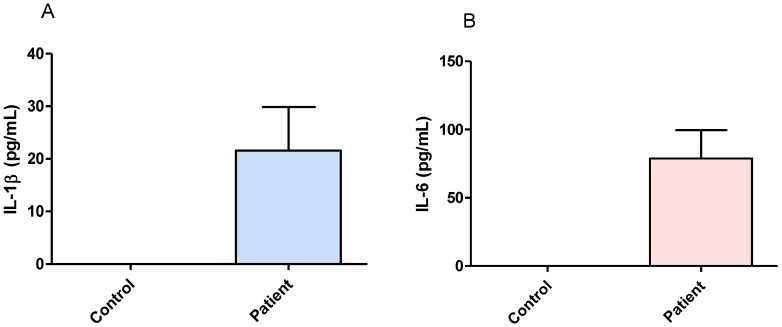
Concentrations of IL-1β and IL-6 in neocortical tissue from patients with drug-resistant temporal lobe epilepsy and control tissue. (**A**) Concentrations of IL-1β. (**B**) Concentrations of IL-6. The bars represent the mean ± the standard error of the mean.

Neocortical tissue from DRTLE patients showed values of IL-1β between 9.69 and 78.32 pg/mL with a mean value of 21.58 pg/mL (Figure 3A), and IL-6 between 17.53 and 278.3 pg/mL with a mean value of 78.87 pg/mL (Figure 3B). The concentrations of both cytokines in the control group presented values below the limit of detection for the method used (Figure 3A,B).

### 3.2. Neocortical Tissue Expression of NF-κB

**Figure 4:**
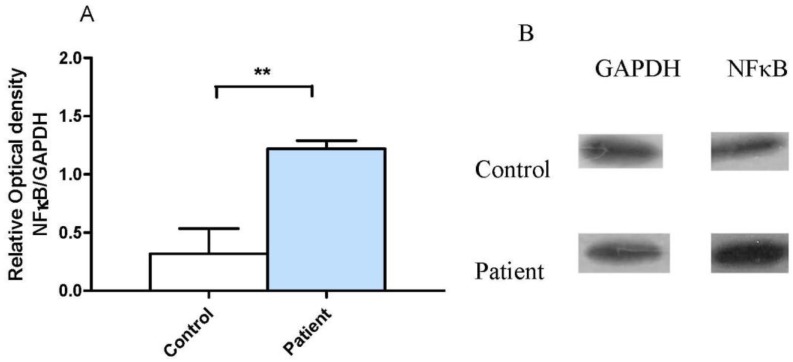
Expression of NF-κB in neocortical tissue of patients with drug-resistant temporal lobe epilepsy. (**A**) Comparison of relative optical density values of NF-κB in patients and control subjects. (**B**) Representative example of the immunodetection of NF-κB in a patient and a control, as well as for GAPDH. The bars represent the mean ± the standard error of the mean, *t*-Student test, ** *p* ≤ 0.001

The presence of the molecule involved in the inflammatory cascade and linked to death processes in neocortical tissue, NF-κB, was evaluated, and the results are shown in Figure 4. Figure 4A shows a statistically significant increase in the expression of NF-κB in the group of patients with DRTLE compared with in the control subjects, while Figure 4B represents an example of the western blot bands in a patient with DRTLE and a control subject, as well as those for GAPDH.

### 3.3. Relationship between Central Inflammation and Apoptotic Neuronal Death

**Figure 5:**
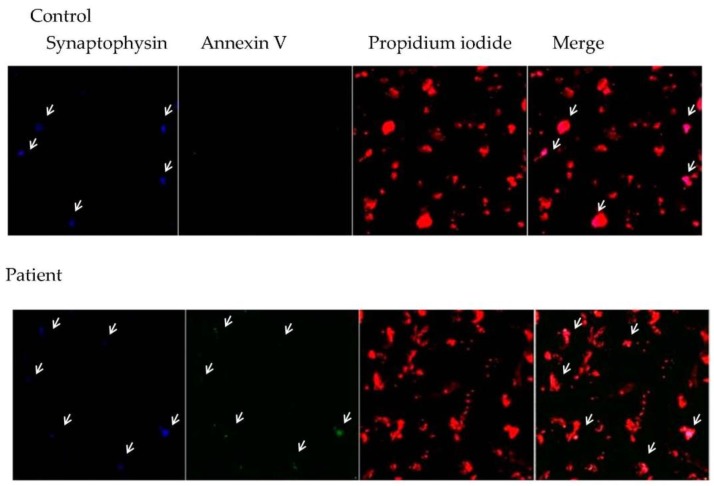
Confocal image of the neocortex of a control subject and a patient illustrating the immunodetection of annexin V + cells (green) doubly marked with synaptophysin (blue) and counterstained with propidium iodide (red). Note that the patient’s annexin V + cells coincide with synaptophysin + (40×).

Figure 5 shows an increase in co-localization of annexin V and synaptophysin positive cells in DRTLE patients in comparison with in control tissue. It is important to note that there is a lower number of positive synaptophysin cells in patients in relation to in the control subjects.

**Figure 6:**
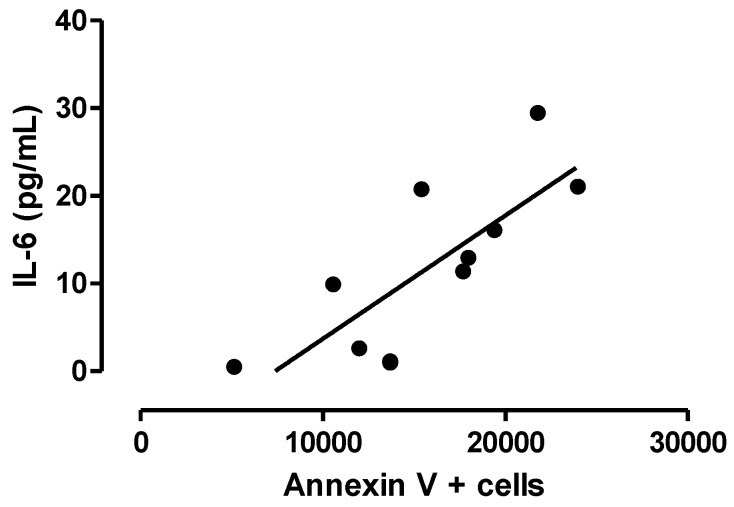
Correlation between the number of annexin V positive cells and IL-6 concentration. Spearman, *r* = 0.8519, *p* ≤ 0.001.

There is a positive correlation between the percentage of immunopositive cells to annexin V and the concentration of IL-6 in neocortical tissue (Figure 6).

## 4. Discussion

### 4.1. Inflammatory Cytokines (IL-1β and IL-6)

The concept of “immunological privilege” of the CNS has been eliminated because of, among other reasons, the accumulation of evidence that immune responses and inflammatory reactions are involved in the pathogenesis of several brain diseases like epilepsy [32]. There is evidence of cerebral inflammation associated with drug-resistant epilepsy of various aetiologies in patients who underwent surgery and whose brain tissues had proinflammatory molecules, reactive astrocytosis, activated microglia, and other indicators of inflammation in the hippocampus [33]. There are several experimental studies that show that the activity of epileptic seizures by themselves can induce cerebral inflammation and that the recurrence of these seizures prolongs chronic inflammation. Similarly, the action of inflammatory mediators on the generation of seizures has been proposed [34,35].

The study of different models of epilepsy developed to evaluate the inflammatory response has shown that, in rodents, with convulsive activity occurs the following molecular cascade: a rapid increase in the proinflammatory cytokines (IL-1β, IL-6, and TNF-α), the activation of the signaling pathway of Toll-like receptors (TLRs), the production of chemokines, activation of the complement system, and increased expression of adhesion molecules [23,25,36,37,38,39].

On the other hand, the literature supports that the immunological and inflammatory alterations described in epilepsy may be associated with anti-epileptic drugs. Changes in inflammatory markers related to carbamazepine, valproate, and other drugs are described [40,41].

Serum levels of IL-1β and IL-6 before and after surgery were evaluated in order to detect if there were changes in the peripheral proinflammatory cytokines from DRTLE patients and if these changes were modified once the epileptogenic zone was resected. This follow-up study was related to the clinical evolution of these patients in terms of the presence or absence of seizures one and two years after surgery. The decrease in the concentration of IL-1β and IL-6 one year after surgery and the fact that in this period most of the patients are free of seizures supports the idea that seizures are the cause of the inflammatory disorders observed in patients with DRTLE. Interestingly, 70% of patients remained seizure-free one year after surgery and the remainder (25%) showed a substantial reduction in seizure frequency.

According to our results, the low levels of IL-6 in patients who were free of seizures one year after surgery in comparison with those in the patients who still experienced them allow us to confirm that this inflammatory marker is closely linked with the occurrence of seizures. Our results indicate that once the epileptogenic zone is resected and seizure activity is reduced, there is a decrease in proinflammatory cytokines. These findings may suggest that seizures are the cause of inflammatory disorders observed in patients with DRTLE.

Other authors report an increase of IL-6 concentrations in patients with epilepsy compared with those in control subjects. They describe this increase associated with the temporal location of the epileptogenic zone [42]. Coincidentally, previous studies by our group showed that alterations in cellular immunity are associated with the location of the epileptogenic zone. The immune alteration was observed in temporal but not in extratemporal localization [43].

An important aspect to assess is the integration of inflammatory events that occur both peripherally and centrally. The existence of several mechanisms by which peripheral inflammation interacts with the brain and induces inflammation at the central level has been discussed. The immediate recognition of inflammatory mediators such as cytokines can occur in circumventricular organs due to the expression of TLRs and IL-R in these structures [44]. The cytokine signal is another mechanism that mediates the response in the brain to peripheral inflammation. Cytokines use both the neural pathway (vagus afferents) and the humoral pathway to communicate the innate immune system to the brain. These mediators can also be transported through the blood–brain barrier (BBB) [45,46,47], and these cytokine signals trigger a central inflammatory response with activation of the microglia, which that induce and propagate these inflammatory signals in the CNS [48].

The evaluation of IL-1β and IL-6 levels in brain tissue (neocortex) of patients with DRTLE showed values that indicate the presence of an inflammatory process at the central level reinforced by the absence of these markers in the control tissue. Tissue obtained at autopsy from subjects with no evidence of neurological disease was used as controls since previous reports indicate that proteins from brain tissue are preserved for several hours after death [28,29,30,49]. Previously, other authors supported the nondetection of concentrations of proinflammatory cytokines in the brain tissue of control subjects [50]. Coincidentally, in resected tissue of patients with TLE, other investigators have suggested that the inflammatory pathways are activated during epileptogenesis and that they persist in epileptic tissue, which contributes to the etiopathogenesis of TLE [34,51].It is also known that inflammatory activity is affected in different ways depending on the severity of the seizures [52,53].

In particular, the analysis of the mRNA of IL-1β and IL-1Ra after the systemic injection of kainic acid in rats shows a significant induction in the microglial cells of the hippocampus, as well as in other areas of the limbic system [54]. Vezzani et al. provided evidence of the rapid increase in IL-1β levels in the hippocampus after seizures in the model with kainic acid; they assume that it occurs as a result of the activation of microglial cells [55]. The increase of IL-1β, IL-6, and TNF-α in the microglia and astrocytes is followed by a cascade of inflammatory events that can recruit cells of the adaptive immune system [33].

Indistinctly in blood or cerebrospinal fluid, an increase in IL-1β and its receptor in astrocytes, microglia, and neurons was documented in a series of patients surgically treated for TLE with hippocampal sclerosis. IL-1β and its receptor are highly expressed in the neurons and glia of patients with TLE caused by focal cortical dysplasia and neuroglial tumors, where expression in brain tissue without these alterations is negligible [34].

In summary, we can affirm that there are high IL-1βand IL-6 concentrations at both the systemic and the central levels that sustain the presence of an inflammatory response in DRTLE. The decrease of both cytokines after surgery in patients with a drastic reduction in seizures supports the idea that these events are an important cause of inflammation.

### 4.2. Expression of Molecules Associated withInflammation (NF-κB) and Apoptotic Death in Brain Tissue from DRTLE Patients

Previous studies report neuronal death in the neocortical tissue of patients with DRTLE [56,57], making it necessary to evaluate the expression of a molecule that participates in the death mechanism by apoptosis and which is linked to inflammatory processes: NF-κB.

Increased expression of NF-κB in the neocortical tissue of patients with DRTLE was observed. This fact confirms the activation of the inflammatory response at the central level and helps to explain the presence of apoptotic neuronal death observed in this study.

NF-κB is a key mediator of the innate and inflammatory immune response; it is activated by inflammatory cytokines through the TNF receptor, IL-1 receptor, and the TLRs family, and, in turn, their activation gives rise to the expression of other cytokines, proteases, and metabolic enzymes [58].

NF-κB has been shown to be involved in the neuropathological processes associated with seizures in epilepsy. Epileptic seizures have been shown to trigger a rapid increase in the amount of NF-κB activated in the hippocampus [59] and its over-expression has been demonstrated in reactive astrocytes and surviving neurons of patients with hippocampal sclerosis [60].

There are contradictory criteria related to the existence or otherwise of other affected areas different to the mesial regions in DRTLE [61]. Previous results from our work group support neuronal death in layer IV of the neocortex adjacent to the hippocampus. This layer is made up of gabaergic interneurons, neurons that are especially sensitive to damage, and glutamatergic-type afferents that explain the occurrence of death processes due to excitotoxicity [62].

Annexin V is described as an early marker of apoptotic processes. Once the process of apoptosis in a cell is activated, the externalization of phosphatidyl serine that is distributed asymmetrically in the cell membrane is produced and is able to bind annexin V. In our study, increased immunoreaction to coincidental annexin V was detected with immunodetection to synaptophysin in the neocortex of patients with DRTLE, which confirms the presence of neurons that initiate the apoptotic process in this tissue.

The neuronal death observed in our results is probably due to the inflammatory process and to the involvement or mitochondrial dysfunction caused by the depolarization of the membrane and the loss of permeability of the mitochondrial membrane, a fact that usually implies the death of the cell [63]. Prolonged exposure to reactive oxygen species can cause depolarization of the mitochondrial membrane and subsequently result in an alteration of the permeability of this structure. Our working group has previously described the presence of an imbalance of the redox system in this type of patient [64]. Another possibility could be the release by the mitochondria of the inhibiting factor of apoptosis that can directly cause chromosomal damage and/or the reactive oxygen species of lysosomal cathepsins that can also provoke mitochondrial damage [63].

The relevant role of apoptotic death in epilepsy has been affirmed by other groups [65,66,67]. Some authors state that neuronal loss after epileptic seizures may be due to an active mechanism of apoptotic death, due to the finding that different classes of regulatory proteins in this process are activated by seizures, including caspases, death receptors, and proteins. The family of Bcl-2, DNA fragmentation pattern, and structural changes all predict apoptotic cell death (Graham [63,68,69,70,71]).

In summary, our results show the increase in immunodetection with annexin V and the existence of a correlation between the concentration of IL-6 and the percentage of annexin V positive neurons in patients with DRTLE, which speaks in favor of the occurrence of an apoptotic neuronal death process. Similarly, we have shown an increase in the expression of NF-kBas well as the cytokines IL-1β and IL-6, and immunodetection of annexin V co-expressed with positive synaptophysin cells. All these findings support the occurrence of inflammatory and apoptotic processes in DRTLE patients.

## 5. Conclusions

The evaluation of the participation of inflammatory processes in DRTLE is aimed at answering one basic question: Is inflammation part of the etiopathogenic processes in DRTLE or are the seizures the result of inflammatory disorders? In this sense, our results describe the clinical evidence that once the epileptogenic tissue has been resected with consequent elimination of seizures (or a significant reduction of them), the observed inflammation disappears. This finding supports the hypothesis that the inflammation could be mostly the consequence of epileptogenic processes. Similarly, this study supports the criterion of the involvement of inflammation and the activation of pathways such as NF-κB, and the final occurrence of death processes of the neuronal population is represented.

All these results highlight the necessity for new research about the role of inflammation and the immune response in the CNS, particularly in DRTLE, in order to achieve an understanding of the epileptogenic mechanism in this clinical entity and open up new possible immunomodulator treatments, in particular for those cases in which surgery is not a therapeutic alternative.

## Figures and Tables

**Table 1:** Summary of clinical data from control subjects and postmortem interval.

Control	Cause of Death	Postmortem Interval (h)
1	Pulmonary thromboembolism	2.5
2	Vehicle accident	3
3	Vehicle accident	4
4	Vehicle accident	3
5	Vehicle accident	3.5

**Table 2:** Clinical data from patients with drug-resistant temporal lobe epilepsy submitted to temporal lobectomy.

Patient Number	Age (Year)	Gender	Personal Pathological History	Side of Focus (R, L)	Disease Duration	Pre-Surgery Number of Seizures per Month	Engel Scale	
							One Year Post-Surgery	Two Years Post-Surgery
1	23	F	Bronchial asthma	L	16	11	IIIA	IIIA
2	41	F	No history	L	15	15	IIIA	IIIA
3	35	F	Febrile seizures	R	35	12	IA	IA
4	26	F	Cranioencephalic trauma	R	11	13	IA	IA
5	35	M	Meningoencephalitis	R	34	12	IA	IA
6	31	M	Febrile seizures	R	30	10	IA	IA
7	41	M	Bronchial asthma	L	36	21	IA	IA
8	26	F	Febrile seizures	L	26	15	IIA	IIIA
9	36	M	Cranioencephalic trauma	R	32	12	IA	IA
10	37	F	Bronchial asthma	R	21	11	IA	IA
